# Elements associated with Iranian women's sexual behaviours: A cross-sectional study

**Published:** 2018-05

**Authors:** Effat Merghati Khoei, Tahoora Alavi, Raziyeh Maasoumi, Fatemeh Sheikhan

**Affiliations:** 1 *Community-Based Participatory Research Center, Iranian Institute for Reduction of High-Risk Behaviours, Tehran University of Medical Sciences, Tehran, Iran. *; 2 *Brian and Spinal Cord Injury Research Center (BASIR), Neuroscience Institution, Tehran University of Medical Sciences, Tehran, Iran.*; 3 *Islamic Azad University, Qazvin Branch, Qazvin, Iran.*; 4 *Department of Midwifery, Khalkhal Branch, Islamic Azad University, Khalkhal, Iran.*

**Keywords:** Sexual behaviour, Iranian women, Reproductive age

## Abstract

**Background:**

Women constitute about half of the Iranian population. Sexual behaviour is one of the most important elements in their lives. Identifying the elements associated with sexual behaviours seems necessary in order to draw a thorough picture of Iranian women's sexuality.

**Objective:**

To elicit information from Iranian women at their reproductive ages on sexual behaviours related to their elements including sexual capacity, sexual motivation, performance and sexual scripts.

**Materials and Methods:**

Study participants involved 295 women at reproductive age from five different cities. Women completed a national self-reporting questionnaire on elements related to sexual behaviours. The elements included sexual capacity, sexual motivation, sexual performance, and sexual script. Pearson’s correlation variance analysis and multi-linear regression were used to analyze data.

**Results:**

Significant positive correlation was found between the sexual capacity, motivation, performance, and sexual script (p<0.001). Linear regression showed that the effective variable on the sexual performance were women's ages (p=0.02), and tertiary education (p=0.05). A significant association was found between age and sexual motivation score, too. A significant relation was observed between the history of pregnancy and level of education with a positive response to sexual script questions**.**

**Conclusion:**

Identifying the elements of sexual behaviours would help women understand their sexual behaviours and related influencing factors. Therefore, enrichment of women's sexuality is needed; also a well-planned educational program is a need for women to understand their sexuality-related potentials.

## Introduction

women constitute about 49.6% of Iran population and 71% of total population are at age 15-65 yr. Iranian women are living in an environment that is significantly different in terms of sexualization (1-4). Sexual role includes the way someone acts, feels and attitudes toward being a male or female or the gender role expectations (2). Reproductive age is a period when a person becomes sexually active with or without marriage (5). The quality of one's sexual life is influenced by various factors related to sexual behaviours (1, 6). 

In spite of being innate and spontaneous, sexual behaviours are subject to be learned; therefore, sexual performance may have described differently within a society and even the formation of sexual behaviour for a person changes from time to time (7). Sexual behaviour is a complex concept. Hilbert, a contemporary sexologist, defines human sexual behaviour based on three factors: sexual capacity (what one can do), sexual motivation (what one wants to do), and performance (what one does) (2).

There is numerous agents influence constructing human sexual behaviours, such as biological agents (anatomy and physiology of genital organs) psycho-cognitive agents (sexual intelligence, body image, in sight of a person toward sexual affairs) and socio-cultural agents (sexual affairs and gender-related issues). In other words, human sexual behaviours include all aspects of body, life experiences, knowledge, mind, and spirituality (8). 

Construction and formation of sexual behaviours are different in various cultures. Some sexual behaviours are the interest of a person and are acceptable by the society, while some of them are considered inappropriate (9). It does not mean that unacceptable behaviours are not performed in interpersonal relationships. Some of the sexual behaviours are negotiated between sexual partners even they are culturally intolerable (10, 11). 

Divorce rate increased 11.5% in 2013 in Iran. Some reports show that 60% of divorces’ roots are in sexual affairs. Hence, the emergence of the problem; sexuality disorders and their negative potential effects on couples' sexual satisfaction, and ultimately the quality of life among couples is a hypothesis to investigate sexual behaviours of women as the largest proportion of Iranian population (1).

There is a little information available from Iranian women's sexual lives and the related influencing factors. A survey titled “relation between sexual satisfaction, matrimony satisfaction and the couple life satisfaction” reported a meaningful relation between sexual satisfaction and matrimony satisfaction as well as matrimony and life satisfaction (12). Another study found that the gender role scripts in women were related to fulfilled sexual behaviour (13). Study with 306 Iranian married women at their reproductive age reported emotional bond as the most important attribution of women's sexual satisfaction (14). 

However, to the best of authors' knowledge, none of the studies conducted in the Iranian contexts has analyzed the influencing elements on women's sexual behaviours. This report is based on findings from the secondary analysis of the data gathered to identify knowledge, attitude and practice of Iranian women at their reproductive age. 

## Materials and methods

In this cross-sectional study, 295 women at their reproductive ages were recruited from five different cities. The study criteria were women at age 15-49 yr, literate, sexually active in the past 30 days, and lack of any known physical or mental problem under treatment. The sample size was calculated based on 95% confidence coefficient; the maximum acceptable error was 2% and possible loss rate of 20%. Convenient sampling was conducted throughout the educational workshops named: “enhancement of marital relationship” held in cities of Tehran (Capital of Iran), Qom (the eighth largest city in Iran, in the southwest of Tehran), Zabol (in the southwest of Iran), Bandar Abbas (in the south of Iran, Capital of Hormozgan Province) and city of Khoy (the west of Azarbayijan Province). 

These workshops were sponsored and held by the authorized organizations such as City Council, Medical Sciences Universities, Islamic Azad Universities, and Non-Governmental Organizations. These programs were offered in three-day (12 hr) workshop by the first author as a sexologist. The subjects voluntarily participated and completed two questionnaires: demographic questions including age, level of education (woman and spouse), marital status, occupation (woman and spouse), pregnancy, number of children, economic status, disease, drug consumption and medicine, and 33-item instruments evaluating women sexual behaviours in their reproductive ages. This questionnaire had been developed and validated by Ghorashi, Merghati Khoei, and Yousefi (2014) in a large mixed methods research (15). For the reliability of this questionnaire, Cronbach’s alpha coefficient was reported to be 0.81. This instrument examines sexual capacity (Q1-10), sexual performance (Q11-19), sexual motivation (Q20-30), and sexual script (Q31-33). 

Each question was scored between 0-5 (0= I don’t know, 5=very much). Prior to the start of educational classes, questionnaires were filled out by the participants and put in a closed and safe box. Women were recruited from different cities based on the range of women attended the class: Tehran 27.9%, Qom 11.1%, Bandar Abbas 4%, Zabol 19.8%, and Khoy 37.2%.

**Ethical consideration**

The study protocol was approved by the ethics committee of Community-Based Participatory Research Center at Tehran University of Medical Sciences, Tehran, Iran (code: 92-03-62-23871). Informed consent was obtained at the initial visit with additional permission attained to use result anonymously in future studies.

**Statistical analysis**

Data analysis was completed using SPSS software (Statistical Package for the Social Sciences, version 20.0, SPSS Inc, Chicago, Illinois, USA) for Windows using descriptive-statistical tests, Pearson’s correlation, variance analysis, Tukey, Chi-square and multiple linear regressions. The significance level was set at p=0.05.

## Results

The mean and standard deviation of the participants’ age was 35±7.3 and 38±7.8 yr for their spouses. There was a significant statistical difference among the participants’ age mean in different cities. Based on the results from Tukey test, the mean and standard deviation of the samples’ age in Tehran (31.1±4.5) had a considerable statistical difference with Bandar Abbas (44.2±7.3), and Khoy (38.9±7.8) (p<0.001). Majority of women who participated in the survey were married (97.5%), employed (44.4%) and had a tertiary education (85.3%). Among married participants, 7.6% had three children or more, 39% had two children, 46.2% had one child, and 7.2% of them had no child. 

**Figure 1:**
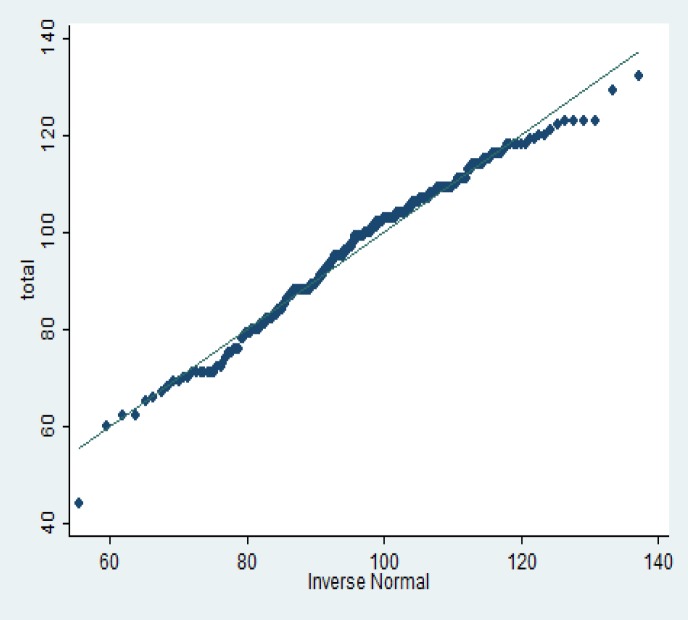
Normal distribution scores of samples, sexual behaviours

Chi-square test results showed that there was a significant association between the number of children and education. In other words with increasing level of education, the number of children was significantly decreased (p<0.001); while Kruskal-Wallis nonparametric test results showed that there was not a significant association between the number of children and people’s economic and social status (p=0.46). Moreover, Swilk normality test results showed that variable distribution of sexual behaviours’ total score was normal (Figure 1).

**Figure 2:**
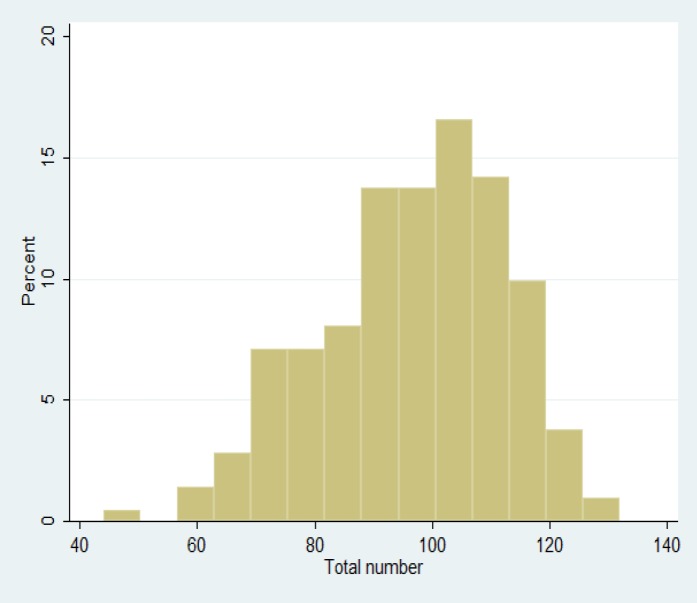
Participants’ sexual behaviours total score

Based on the results of table II, the normal range of sexual behaviour score was considered from 62 to 124 and 50^th^ percentile or mean score of the samples sexual behaviours was 97. It illustrated that 50% of participants scored under 97, and 50% of them gained a score above 97. The mean and total standard deviation of sexual coefficient variables was 96.3±15.7. The participants’ sexual behaviours total score was between 44 and 132 with the mean about 99 (Figure 2). The analysis of the total score of sexual behaviours showed that the only effective variable on the sample’s sexual behaviours was the age that it decreased by the coefficient of 0.44 as age unit (yr) increased.

The results of regression analysis showed that there was not a significant statistical relation among a mean score of sexual capacity and age, education, occupation and people’s economic status. Although sample’s ages from different cities were not the same, results of linear regression analysis indicated that this dissimilarity has not had any impacts on the participants’ sexual capacity. Mean and standard deviation for the 9th question (my sexual desire is more than my husbands) and the 8th question (my desire increase by husband’s affection) from people sexual capacity sub-scale has allocated the least (2.68±1.28) and the most (4.12±1.02) amount to itself.

**Figure 3:**
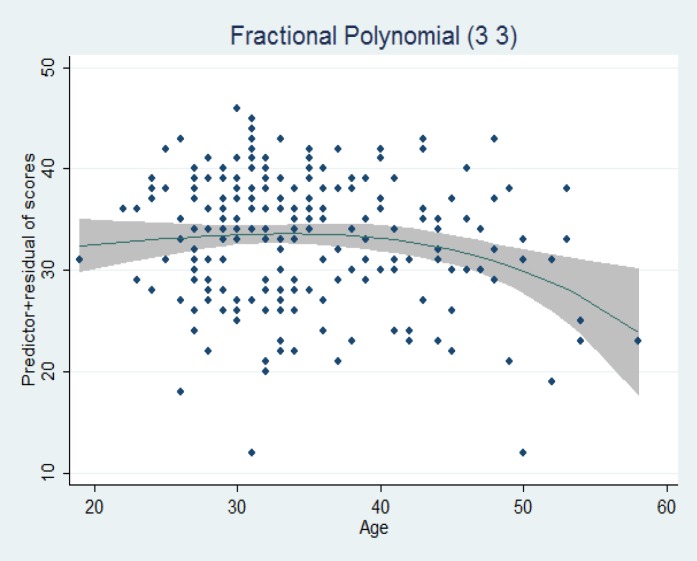
The relation between age and sexual motivation total score (f=1.08

The results of variance analysis showed there was not a meaningful relation between the mean scores of different cities in the area of sexual capacity (p=0.08). In relation to effective variables on the samples, scores of sexual motivation, results of linear regression with the stepwise backward method illustrated that none of the demographic variables have been effective on the samples’ score of sexual motivation. Figure 3 shows the relation between women age and a total score of sexual motivation sub-scale. The results showed that there was a linear relation between age and sexual motivation score that declined by getting older. Results of the linear regression analysis showed that effective variables on sample’s performance score were age (regression standard coefficient of -0.17 and p=0.023) and having a college education (regression standard coefficient of 0.41 and p=0.05). The results from one-way analysis of variance suggested there was a significant statistical difference between mean scores of sexual performance in different cities (p=0.01) (Table I).

Results show that motivational effect of question 2 (I think my husband’s expectation from sexual relation is just his orgasm) was 37.1±2.2, and question 3 (my husband never pays attention to the appropriate time for sex and does not consider my problems) was 46.2±35.1, and question 10 (my husband’s tendency to copy sexy movies and pictures decreases my sexual tendency) was 93.1±46.1. All of these cases were less than other questions. It seemed that lack of husband’s attention to his wife or individualism in his sexual needs decreased the women participants’ sexual motivation. The most and the least mean scores in sexual performance sequentially referred to the first question (53.1±05.3) (I can reach him to maximum sexual enjoyment by oral sex) and the ninth question (26.1±72.0) (I sometimes masturbate). Tukey test results demonstrated that median scores of the participants’ sexual performance in Tehran (19±5.6) had a considerable increase compared to the samples in Bandar Abbas (13.9±6.4) (p=0.01).This difference was not seen in other cities.

Majority of women answered positively to script questions. First question (sexual requests positive response to husband’s leads to reward): religious beliefs 52.2%, second question (Positive response to husband’s sexual requests makes marital connection more sincere and create peace in the family): communicative beliefs 78.1%, third question (Positive response to husband’s sexual requests prevents him from tending to other women): cultural and traditional beliefs 58.5%.

Exploring the sexual script questions, it was indicated that previous pregnancy variables had a significant statistical relation with positive answers to first question p=0.02 and college educational level with a positive answer to the third question (p=0.002). 151 women (61.9%) who answered positively to the third question of the sexual script had a college education. Results from Pearson correlation test showed that there was a positive and meaningful correlation between the score of capacity, motivation, performance and sexual script.

**Table I:** The results of one-way variance analysis test for comparison of samples’ sexual performance mean scores

**Participated cities**	**Mean ± standard deviation**	**Statistic test F**	**p-value**
Tehran	19 ± 5.6	3.37	0.01
Qom	19.2 ± 5.7		
Bandar Abbas	13.9 ± 6.4		
Zabol	16.3 ± 6.5		
Khoy	17.7 ± 6		

**Table II:** Normal range of scores was determined by Xrimli method (percentile between 5.2 and 5.97).

**Variable**			**Observation**			**Mean±SD**	**Min**	**Max**
Ec2-5-ml		298			62.40495±0		62.40495	62.40495
Ec10	298				76.02965±0		76.02965	76.02965
Ec25	298				86.70728±0		86.70728	86.70728
Ec50	298				97.42892±0		97.42892	97.42892
E75	298				107.2602±0		107.2602	107.2602
E90	298				115.5156±0		115.5156	115.5156
E95	298				120.2321±0		120.2321	120.2321
Ec97_5_ml				298	124.2036±0		124.2036	124.2036

Swilk normality test results showed that variable distribution of sexual behaviours’ total score was normal

## Discussion

A positive and meaningful correlation has been shown in this survey between scores of capacity, motivation, performance, and sexual script. According to the results, some of the demographic variables significantly changed the obtained score in sub-scales of capacity, performance, motivation, and sexual script. Analysis results of effective variables on total score on sexual behaviours showed that the only effective variable on sexual behaviour is the age.

According to the present study results, the increase in sexual performance score had a meaningful statistical relation with the decrease in their age and the increase in their education level. Our results are inconsistent with the Merghati Khoei findings exploring marital status and women’s sexual function. They noticed their findings might be due to the emotional support from partners in married women or lack of sexual activity in widows (16). Rahimi and colleagues also noted that women with higher educational level had higher sexual performance scores. They believe that educated women more likely participate in sexual educational programs (17). Consistent with our results, Tomic and coworkers, found there was not significant associations between socioeconomic status and women’s sexual performance (18). In contrast, the results of research on 6029 American married women revealed that sexual activity was reduced by the presence of any children 18 yr of age or younger in the family (19). 

Merghati khoei and colleagues study on 200 middle-aged women (40-65) showed that there was a meaningful statistical relation between age, education level, the economic and social status of samples with their sexual performance indicator. Additionally, the low score of sexual performance had a significant connection with variables of retirement, hysterectomy, oopherectomy, hot flashing, cold sweating, vaginal dryness, illness and drug consumption (16). In this study sexual capacity had not significant relation to age, education and peoples’ economic status. Addis and colleagues found that women’s sexual satisfaction reduced with the age increasing (20). Rahimi and colleagues believe that some tasks including tasks related to children, job interference to sexual activity result to decrease sexual satisfaction with the increase in the age (17). Hendrick and co-workers study results were parallel with ours. They indicated that there was a significant relation between the increases in women’s ages and the decreases in their sexual motivations (21). People’s sexual motivation was influenced by different factors such as hormonal, cultural, cognitional factors, and people’s learning (22). 

Our results in sexual script section demonstrated that women’s sexual behaviours were affected by a sexual script that was affected by religious instructions and traditional attitudes that are partially wrong. According to our results educational level had not a significant statistical relation with positive answer to first script question (religious-sexual question) “sexual requests Positive response to husband’s leads to reward”. Merghati Khoei and coworkers showed that religion is a key factor in women’s sexual self-understanding; in addition they noted sexual obedience has a great impact on women’s chastity and self-steam (23). It seems that women’s employment and educational interventions change women’s sexual behaviours. Rezai and colleagues showed religion had impact on the peoples’ sexual behaviours (24). The second script question “positive response to husband’s sexual requests makes marital connection more sincere and creates peace in the family” is about communicative beliefs. Merghati Khoei and colleagues believe that health care providers should consider emotional connection between couples when they offer health care since it plays an important role in women’ sexual satisfaction (14).

Third question of sexual script “Positive response to husband’s sexual requests prevents him from tending to other women” is a restricted and male-dominated sexual scrip. In our study women with college educational level had positive answer to this question. Hashemi and colleagues considered the issue of obedience and women’s obligation to comply with their husband’s demands as well as women’s fear of their husbands’ extramarital affairs as main reasons why women could not refuse their husbands’ sexual demands (25). In fact, our findings based on the effect of the sexual script on sexual behaviours confirmed social determiners in the formation of sexual behaviours that had a similarity with socio-structuralism theory (9). Results of this study revealed participants’ sexual behaviours decreased by the increase age. In addition there was a linear relation between age and sexual motivation score. The effective variable on sample’s performance score were age and having a college education. The obtained results could be a scientific theme and based on evidence could be used for policy, planning, educational, therapeutically, interference in women’s sexual health in fertility ages.

**Limitations**

A seminal limitation should be noted. Like other cultures, sexuality has been strongly mingled with socio-cultural norms; the supremacy of these factors could limit the utterance of issues by participants in the study. In addition we suggested a same study to investigate the elements associated with Iranian men’s sexual behaviours.

## Note

***This article extracted from M.Sc. Thesis. (Tahoora Alavi)***

## References

[1] Merghati-Khoei E, Solhi M, Nedjat S, Taghdisi MH, Shojaeizadeh D, Taket AR (2014). How a Divorcee’s Sexual Life Is Socially Constructed and Understood in the Iranian Culture. J Divorce Remarriage.

[2] Ghorashi Z, yousefy A, Merghati-khoei E (2016). Developing and validating a questionnaire to measure women’s sexual behaviors: A psycho-metric process. Galen Med J.

[3] Merghati-Khoei E, Qaderi K, Amini L, Korte JE (2013). Sexual problems among women with multiple sclerosis. J Neurol Sci.

[4] Merghati Khoei E, Norouzi Javidan A, Abrishamkar M, Yekaninejad MS, Chaibakhs S, Emami-Razavi SH (2013). Development, validity and reliability of sexual health measures for spinal cord injured patients in iran. Int J Fertil Steril.

[5] Martinez GM, Abm JC (2015). Sexual activity, contraceptive use, and childbearing of teenagers aged 15-19 in the United States. NCHS Data Brief.

[6] Andersen BL, Cyranowski JM (1995). Women's sexuality: behaviors, responses, and individual differences. J Consult Clin Psychol.

[7] Clyton AH, McGarvey EL, Clavet GJ (1997). The changes in sexual Functioning Questionnaire (CSFQ): development, reliability and validity. Psychopharmacol Bull.

[8] Avasthi A, Kaur R, Prakash O, Banerjee A, kumar l, Kolhara P (2008). Sexual behavior of married youngewomen:A preliminary study from north india. Indian J Community Med.

[9] Gagnon J, Simon W (2005). Sexual conduct; the social sources of human sexuality.

[10] Merghati-Khoie E (2015). My Wife.

[11] Masters NT, Casey E, Wells EA Morrison DM (2013). Sexual scripts among young heterosexually active men and women: Continuity and change. J Sex Res.

[12] Hojjat Panah M, Ranjbar Kohan Z (2014). A study of relationship between Sexual Satisfaction, Marital Satisfaction and life satisfaction in Couples. Cheshmandaz Amin Appl Psychol.

[13] Khamsei A (2007). Study of relationship between sexual behavior and gender role stereotypes of women and men in the family. J Family Res.

[14] Merghati Khoei E, Maasoumi R, Talebi S, Hajimirzaie S, Bayat A, Rimaz Sh (2015). Factors Affecting Sexual Satisfaction in Iranian Women. Women's Health Bull.

[15] Ghorashi Z, Merghati-Khoei E, Yousefy A (2014). Measuring Iranian women's sexual behaviors: Expert opinion. J Educ Health Promot.

[16] Merghati-Khoei E, Sheikhan F, Shamsalizadeh N, Haghani H, Yousofnia Pasha YR, Killeen T (2014). Menopause negatively impacts sexual lives of middle-aged Iranian women: a cross-sectional study. J Sex Marital Ther.

[17] Rahmani A, Sadeghi N, Allahgholi L, Merghati-Khoei E (2010). the relation of Sexual satisfaction and demographic factors. Iran J Nurs.

[18] Tomic D, Gallicchio L, Whiteman MK, Lewis LM, Langenberg JA, Flaws JA (2006). Factors associated with determinants of sexual functioning in midlife women. Maturitas.

[19] Donnelly D (1993). Sexually inactive marriages. J Sex Res.

[20] Addis IB, Van Den Eeden SK, Wassel-Fyr CL, Vittinghoff E, Brown JS, Thom DH (2006). Sexual activity and function in middle-aged and older women. Obstet Gynecol.

[21] Hendrickx L, Gijs L, Enzlin P (2015). Age-related prevalence rates of sexual difficulties, sexual dysfunctions, and sexual distress in heterosexual women: results from an online survey in flanders. J Sex Med.

[22] Kelly MJ (1997). Human Sexual Motivation.

[23] Merghati Khoei E, Whelan A, Cohen J (2008). Sharing beliefs: What sexuality means to Muslim Iranian women living in Australia. Cult Health Sex.

[24] Shirpak KR, Chinichian M, Eftekhar H, pourreza A, ramezankhani A (2006). [Sexual health education needs assessment in women referred to family planning health centers in Tehran]. Payesh.

[25] Hashemi S, Khodakarami n, Sedigh S, majd HA, Hasanzadeh SM (2012). [The pattern of sexual behavior in married women]. Payesh.

